# The Neural Oscillatory Basis of Perspective‐Taking in Autistic and Non‐Autistic Adolescents Using Magnetoencephalography

**DOI:** 10.1111/ejn.70109

**Published:** 2025-04-16

**Authors:** Robert A. Seymour, Gina Rippon, Gerard Gooding‐Williams, Hongfang Wang, Klaus Kessler

**Affiliations:** ^1^ Oxford Centre for Human Brain Activity (OHBA), Department of Psychiatry University of Oxford Oxford UK; ^2^ Department of Imaging Neuroscience, UCL Queen Square Institute of Neurology University College London London UK; ^3^ Institute of Health and Neurodevelopment Aston University Birmingham UK; ^4^ School of Psychology University College Dublin Dublin Ireland

**Keywords:** autism, brain oscillations, MEG, perspective‐taking, social cognition

## Abstract

Taking another's perspective is a high‐level mental skill underlying many aspects of social cognition. Perspective‐taking is usually an embodied egocentric process whereby people mentally rotate themselves away from their physical location into the other's orientation. This is accompanied by increased theta‐band (3–7 Hz) brain oscillations within a widespread fronto‐parietal cortical network including the temporoparietal junction. Individuals with autism spectrum conditions (ASC) have been reported to experience challenges with high‐level perspective‐taking, particularly when adopting embodied strategies. To investigate the potential neurophysiological basis of these autism‐related individual differences, we used magnetoencephalography in combination with a well‐replicated perspective‐taking paradigm in a group of 18 autistic and 17 age‐matched non‐autistic adolescents. Findings revealed that increasing the angle between self and other perspective resulted in prolonged reaction times for the autistic group during perspective‐taking. This was accompanied by reduced theta power across a wide network of regions typically active during social cognitive tasks. On the other hand, the autistic group showed greater alpha power decreases in visual cortex compared with the non‐autistic group across all perspective‐taking conditions. These divergent theta and alpha power effects, coupled with steeper response time slopes, suggest that autistic individuals may rely more on alternative cognitive strategies, such as mental object rotation, rather than an egocentric embodied approach. Finally, no group differences were found when participants were asked to track, rather than take, another's viewpoint, suggesting that autism‐related individual differences are specific to high‐level perspective‐taking.

## Graphical abstract

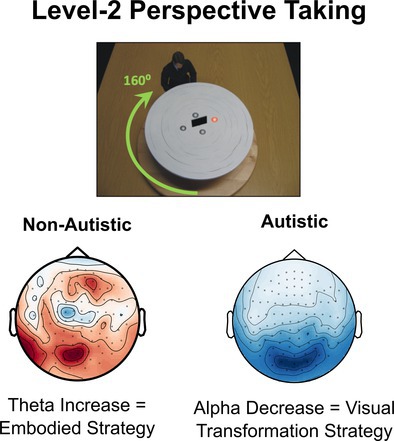

**Abbreviations**
ACC: anterior cingulate ortex
AQ: Autism Spectrum Quotient
ASC: autism spectrum condition
ASD: autism spectrum disorder
DA: directed asymmetry
DAI: directed asymmetry index
EEG: electroencephalography
FIFF: Functional Imaging File Format
GSQ: Glasgow Sensory Questionnaire
HCPMMP: Human Connectome Project Multi‐Modal Parcellation
HPI: head position indicator
LCMV: Linearly Constrained Minimum Variance
LR: left/right (in the context of perspective‐taking trials)
MEG: magnetoencephalography
MNI: Montreal Neurological Institute (brain coordinate system)
MRI: magnetic resonance imaging
PAC: phase‐amplitude coupling
pTPJ: posterior temporo‐parietal junction
RT: reaction time
SQUID: Superconducting Quantum Interference Device
TACS: Transcranial Alternating Current Stimulation
TFR: time‐frequency representation
TMS: transcranial magnetic stimulation
V1: primary visual cortex
V4: Visual Cortex Area V4
VO: visible/occluded (in the context of perspective‐tracking trials)
VPT: visuospatial perspective‐taking
VPT‐1: Level 1 visuospatial perspective‐taking
VPT‐2: Level 2 visuospatial perspective‐taking

## 1 Introduction

Humans possess highly developed social skills that allow us to imagine what others might be experiencing, thinking or feeling to an extent not shared by other species (Tomasello et al. 2005). One important aspect of this is the ability to understand another's visuospatial experience of the world—a skill termed visuospatial perspective‐taking, VPT (Flavell et al. 1981). In this study, we used magnetoencephalography (MEG) to investigate individual differences in the neurocognitive basis of perspective‐taking between autistic adolescents and age‐matched non‐autistic participants.

Considering another's visuospatial perspective of the world is often separated into two levels (Flavell et al. 1981). One can track another's view, termed Level 1 perspective‐taking, VPT‐1. This is often tested by visibility judgements—asking whether another person can see an object or not. Alternatively, one can understand *how* the world looks from another's point of view, also termed Level 2 perspective‐taking, VPT‐2. This is typically measured by asking participants how a visual scene would appear from another's visual perspective, for example, ‘is the object to the left or right from the other's viewpoint?’ (Michelon and Zacks 2006; Hamilton et al. 2009; Kessler and Rutherford 2010; Samuel et al. 2024). The ability to perform perspective‐tracking emerges around age 2, whereas perspective‐taking emerges around ages 4–5 (Flavell et al. 1981; Gzesh and Surber 1985; Moll and Tomasello 2006) and appears to be uniquely human (Karg et al. 2016).

There is growing evidence that perspective‐taking (VPT‐2) typically recruits embodied cognitive processes, grounded in the internal bodily and action representations of the observer. In other words, humans mentally rotate their own embodied self into another's orientation and perspective. This has been shown behaviourally using carefully controlled posture manipulations (Kessler and Rutherford 2010; Kessler and Thomson 2010; Surtees et al. 2013). Furthermore neuroimaging studies have shown that perspective‐taking engages neural regions involved in body schema—the brain's internal representation of the body's position and movement (Gooding‐Williams et al. 2017; Wang et al. 2016; Coslett et al. 2008; Medina et al. 2009). Importantly these embodied processes seem to be specific to perspective‐taking (VPT‐2) but not tracking (VPT‐1), which involves a ‘simpler line of sight’ mechanism (Kessler et al. 2014; Kessler and Rutherford 2010; Martin et al. 2020; Zacks and Michelon 2005); for a recent discussion, see Samuel et al. (2024).

Modern brain imaging techniques have also helped elucidate the neural correlates of embodied perspective‐taking beyond motor regions. Using MEG, Wang et al. (2016) investigated embodied simulation during perspective‐taking using a well replicated paradigm (Kessler and Rutherford 2010). In contrast to perspective‐*tracking* increases in low‐frequency theta‐band oscillations (3–7 Hz) were found for both the cognitive effort (amount of angular disparity between self vs. other's viewpoint) and for embodied processing (posture congruence) during perspective‐*taking*. These effects converged in the right posterior division of the temporo‐parietal junction (pTPJ)—a key hub of the social brain network involved in representing other's mental states (Bögels et al. 2015; Schurz et al. 2013; Van Overwalle 2011). Right pTPJ's involvement was further corroborated by brain stimulation data (Wang et al. 2016; Gooding‐Williams et al. 2017; Martin et al. 2020) and in a follow‐up MEG study using the same paradigm (Seymour et al. 2018). Seymour et al. (2018) showed connectivity changes between the pTPJ and a wide network of brain regions, including (i) social brain regions like the ventromedial prefrontal cortex and medial parietal cortex; (ii) executive control regions such as the anterior cingulate cortex (ACC) and lateral pre‐frontal cortex; and (iii) regions coding for body schema. These findings were specific to theta (3–7 Hz) oscillations suggesting this could be a crucial frequency for embodied perspective‐taking; a hypothesis supported by complementary theta‐specific TMS stimulation (Gooding‐Williams et al. 2017). Overall there is emerging evidence that perspective‐taking is underpinned by an embodied cortical network centred on the right TPJ, while also including the wider social brain network, body schema and executive control regions, coordinated via theta rhythms (Seymour et al. 2018).

In this study we investigated the neural basis of perspective‐taking in participants diagnosed with autism spectrum disorder—a neurodevelopmental condition characterised by difficulties in social interaction, language, repetitive behaviours and sensory sensitives (American Psychiatric Association 2013). Research in this area has produced heterogeneous results, with some studies reporting reduced perspective‐taking accuracy in autistic children (Baron‐Cohen 1989; Hamilton et al. 2009; Yirmiya et al. 1994), whereas others suggest performance on‐par with non‐autistic control participants (Hobson 1984; Tan and Harris 1991; Leslie and Frith 1988). Reviewing the literature, Pearson et al. (2013) suggest that overall, autistic participants display a selective impairment for embodied perspective‐*taking* (VPT‐2), but not perspective *tracking* (VPT‐1).

Impaired perspective‐taking may relate to so‐called ‘mindblindness’ theories of autism (Baron‐Cohen 1997), which describe difficulties in understanding others' thoughts and feelings through theory of mind. The processes of embodied perspective‐taking and theory of mind share a common neural substrate (Apperly and Butterfill 2009; Schurz et al. 2015)—both involve inhibiting our own perspective in favour of considering another's perspective, be that a visual object or another's thoughts and feelings (Hamilton et al. 2009). Impaired visual perspective‐taking could therefore impact the development of social skills in autism more generally (Hamilton et al. 2009; Pearson et al. 2016; Surtees et al. 2013), raising the intriguing possibility that perspective‐taking interventions in autism may transfer into beneficial outcomes for high‐level social and cognitive development (Pearson et al. 2016).

Instead of embodied processes autistic participants may favour alternative compensatory strategies. For instance, Pearson et al. (2016) suggest autistic participants use a mental object rotation strategy during perspective‐taking, mentally rotating the world towards their own perspective (allocentric rotation; Shepard and Metzler 1988). Alternatively, autistic participants may opt for a visuospatial transposition in form of ‘my left is their right’ at high angular disparities (‘spatial transposers’, Gardner et al. 2013; Kessler and Wang 2012). Such alternative strategies might appeal to autistic individuals because processing is essentially egocentric, where the self does not have to be mentally manipulated into another's orientation to understand their perspective. For example, a spatial transposition offers a ‘short‐cut’ where the egocentric left/right is flipped and mapped onto the target perspective. This aligns with suggestions of a stronger egocentric bias in autism (e.g., Baron‐Cohen 1997; Lombardo and Baron‐Cohen 2010, 2011).

These alternative strategies (mental rotation and spatial transposition) lead to the correct answer, but can be more cognitively demanding than embodiment, resulting in longer reaction times and decreased accuracy (Kessler and Thomson 2010). In support of this idea, Kessler and Wang (2012) reported that participants with higher levels of autistic traits, but not diagnosed with autism, showed reduced embodiment effects, compared to participants with lower levels of autistic traits. Accordingly, Brunyé et al. (2012) also showed increased response times for participants with stronger autistic traits. While impaired embodied perspective‐taking in autism has been reported behaviourally (Pearson et al. 2013), no study to date has investigated the oscillatory neural correlates of perspective‐taking and perspective‐tracking in autism.

The current study aimed to investigate autism‐related differences in the neurocognitive mechanisms underlying perspective‐taking, using MEG and a well‐replicated perspective‐taking paradigm (Kessler and Rutherford 2010). We opted to collect data from adolescent participants as social skills, in particular, high‐level perspective‐taking, are still developing during adolescence (Dumontheil et al. 2010) making it an interesting developmental timepoint to study autism‐related group differences. Furthermore, adolescent participants are generally more compliant than children, which is an important consideration given cryogenic MEG data collection requires participant movement to be less than ~5 cm.

First, it has been suggested that perspective‐taking difficulties in autism are specific to perspective‐taking (VPT‐2, Pearson et al. 2013, 2016); thus, we hypothesised no significant oscillatory differences in perspective‐tracking (VPT‐1) between autistic and non‐autistic groups. It was hypothesised that the non‐autistic adolescent group would adopt an embodied egocentric strategy during perspective‐taking, resulting in increased theta‐band power (3–7 Hz) within the TPJ, prefrontal executive control regions and regions coding for body schema, as per Wang et al. (2016) and Seymour et al. (2018). Given theories of perspective‐taking in autism (Pearson et al. 2013), we hypothesised reduced theta power in these regions for the autistic group. Task‐related changes in the alpha‐band (8–12 Hz) were also examined for several reasons. First, perspective‐taking requires active visual processing which is known to involve modulations of alpha‐band power in the occipital lobe (Jensen and Mazaheri 2010; Klimesch 1999). Presentation of the stimuli used in this study are known to robustly elicit alpha‐band desynchronization (Seymour et al. 2018; Wang et al. 2016). Second, Wang et al. (2016) found modulation of alpha rhythms during perspective‐taking when investigating the effect of angular disparity. Third, given that the autistic group are more likely to adopt a mental rotation or transposition rather than embodied strategy (Gardner et al. 2013; Kessler and Wang 2012; Pearson et al. 2016), we hypothesised that the increased visual processing demands would result in greater alpha‐related desynchronization versus the control group (Gardony et al. 2017; Klimesch et al. 2003; Riečanskỳ and Katina 2010). This hypothesis was additionally motivated by neurostimulation research showing enhanced mental object rotation performance during alpha‐band repetitive TMS (Klimesch et al. 2003), as well as alpha‐specific TACS stimulation (Kasten and Herrmann 2017). While embodied and visual processing strategies might both predict modulation of alpha oscillations, we expect that the primary involvement of either theta or alpha, respectively, will be indicative of which strategy was employed. In other words, a strong theta modulation by angular disparity alongside a negligeable alpha effect would indicate the use of an embodied strategy, while a primary alpha modulation alongside a negligeable theta effect would indicate a strategy focused on visual processing (mental rotation or spatial transposition).

## 2 Materials and Methods

### 2.1 Participants

Data were collected from 18 autistic participants and 17 age‐matched non‐autistic controls, see Table 1. We refer to the autistic participants as the autistic spectrum condition (ASC) group based on terminology guidance (Kenny et al. 2016). MEG data from four of these participants (two ASC and two control) were excluded based on RT results (see Section 2.3). Autistic participants had a confirmed clinical diagnosis of Autism Spectrum Disorder or Asperger's syndrome from a paediatric psychiatrist. Participants were excluded if they were taking psychiatric medication or reported epileptic symptoms. Non‐autistic participants were excluded if a sibling or parent was diagnosed with autism. Between groups there were significant higher AQ and GSQ scores, *p* < 0.05, scores for the autistic group. There were no significant differences between groups in age, raven matrices assessment of cognitive reasoning (Raven 1998) or the Mind In the Eyes test (Baron‐Cohen et al. 2001). No members of the non‐autistic control group had an AQ score about 35, which is the cutoff value for clinically relevant autistic traits.

**TABLE 1:** Participant demographic and behavioural data. * = behavioural scores significantly greater in autistic > non‐autistic group, *t* test, *p* < 0.05.

	*N*	Age	Male/female	Autism quotient (adult)	Raven Matrices Score	Glasgow Sensory Score	Mind in the Eyes Score
Autistic group	18	(Range: 13–20) M: 16.67	14 male; 4 female	32.6*	43.8	65.3*	21.8
Comparison group	17	(Range: 14–19). M: 16.41	14 male; 3 female	10.71	48.8	38.4	25.5

All experimental procedures complied with the Declaration of Helsinki and were approved by the Aston University, Department of Life & Health Sciences ethics committee. Written consent was obtained from participants aged 18 or over, or a parent/guardian for participants aged under 18.

### 2.2 Experimental Paradigm and Design

**FIGURE 1:**
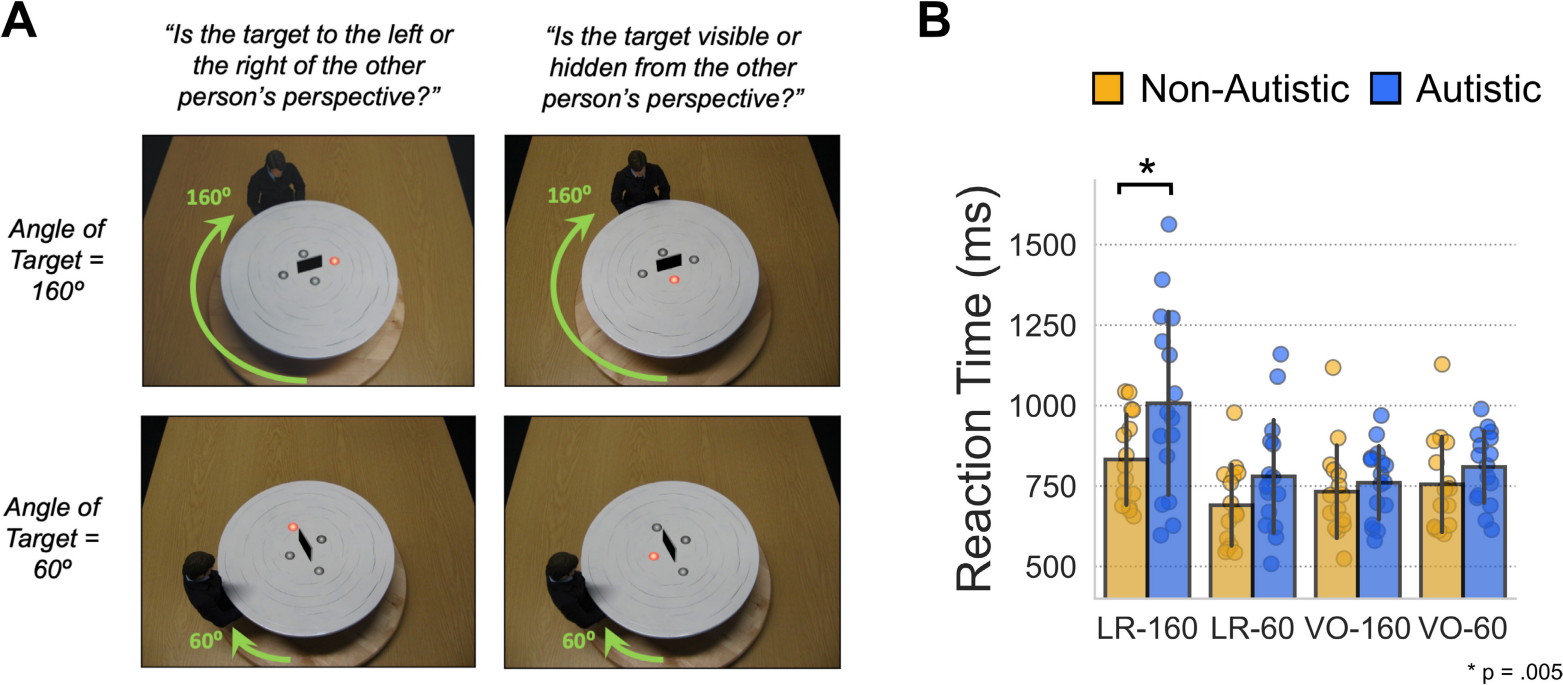
(A) Example stimuli from the paradigm, for each of the four experimental conditions. Arrows are for illustrative purposes and were not presented to participants. (B) Reaction time (RT) data are shown for each of the four experimental conditions. Individual datapoints indicate participant RT medians per condition. Significant differences, *p* < 0.05, between groups were found for the LR‐160 condition.

The paradigm was adapted from Kessler and Rutherford (2010). The stimuli were coloured photographs (resolution of 1024 × 768 pixels), showing an avatar seated at a round table from one of four possible angular disparities (see Figure 1A). In each trial, one of the grey spheres on the table turned red indicating this sphere as the target. From the avatar's viewpoint, the target could be either visible/occluded (VO) by a centrally presented black screen (perspective‐tracking); or to the left/right (LR) (perspective‐taking). Stimuli were presented in 12 mini‐blocks of 32 trials, alternating between LR and VO conditions. On each trial, participants were asked to make a target location judgement according to the avatar's perspective by pressing the instructed key on an MEG‐compatible response pad: the left key for ‘left’ or ‘visible’ targets from the avatar's viewpoint and the right key for ‘right’ or ‘occluded’ targets (see Figure 1A). Accuracy feedback was provided after each trial in the form of a short tone. As in Kessler and Rutherford (2010), we collapsed across clockwise and anticlockwise disparities, and separately collapsed correct responses for left and right and visible and occluded, respectively. This resulted in four separate experimental conditions (e.g., see Figure 1A): left/right judgements where the avatar is 160° from own perspective (LR‐160); left/right judgements where the avatar is 60° from own perspective (LR‐60); visible/occluded judgements where the avatar is 160° from own perspective (VO‐160); visible/occluded judgements where the avatar is 60° from own perspective (VO‐60). This 2 × 2 design allowed us to disentangle perspective‐taking from perspective‐tracking and investigate the effect of an increased angular disparity, which has been shown to lengthen reaction times for perspective‐taking but not for perspective‐tracking (Kessler and Rutherford 2010; Michelon and Zacks 2006). We chose to use 160° versus 60° based on the results of Seymour et al. (2018), Gooding‐Williams et al. (2017) and Wang et al. (2016).

### 2.3 Behavioural Data Analysis

All trials containing incorrect answers or response times greater than 2 standard deviations from the participant's individual mean reaction times were excluded from subsequent analyses. Median RT per condition from each participant, as per Kessler and Rutherford (2010), were entered into a 2 × 2 × 2 mixed ANOVA with condition (LR, VO) and angle (160°, 60°) as repeated measures factors and group (ASC, control) as a between‐subjects factor, using the JASP statistics package. Data from four participants (two ASC and two control) were discarded due to a median RT greater than 2 standard deviations from the group median.

### 2.4 MEG and Structural MRI Acquisition

MEG data were acquired using a 306‐channel Neuromag MEG scanner (Vectorview, Elekta, Finland) made up of 102 triplets of two orthogonal planar gradiometers and one magnetometer. All recordings were performed inside a magnetically shielded room at a sampling rate of 1000 Hz. Five head position indicator (HPI) coils were applied for continuous head position tracking, and visualised post‐acquisition using an in‐house Matlab script. For MEG‐MRI coregistration purposes three fiducial points, the locations of the HPI coils and 300–500 points from the head surface were acquired using the integrated Polhemus Fastrak digitizer. Visual stimuli were presented on a projection screen located 86 cm from participants, and auditory feedback through MEG‐compatible headphones. Data acquisition was broken down into three sequential runs, each lasting 8–10 min.

A structural T1 brain scan was acquired for source reconstruction using a Siemens MAGNETOM Trio 3T scanner with a 32‐channel head coil (TE = 2.18 ms, TR = 2300 ms, TI = 1100 ms, flip angle = 9°, 192° or 208° slices depending on head size, voxel‐size = 0.8 × 0.8 × 0.8 cm).

### 2.5 MEG Preprocessing

All MEG data were pre‐processed using Maxfilter (temporal signal space separation, 0.9 correlation), which supresses external sources of noise from outside the head (Taulu and Simola 2006). To compensate for head movement between runs, data from runs 2 and 3 were transformed to participant's head position at the start of the first block using the *‐trans* option of Maxfilter. For each participant, the entire recording was band‐pass filtered between 0.5–250 Hz (Butterworth filter) and band‐stop filtered to remove residual 50‐Hz power line contamination and its harmonics. Data were then epoched into segments of 2.5 s (1 s pre‐stimulus, 1.5 s post‐stimulus onset), and each trial was demeaned and detrended. Trials containing artefacts (SQUID jumps, eye‐blinks, head movement) were removed by visual inspection, resulting in removal of an average of 86.5 trials per condition, per participant. There were no significant group differences in the number of good trials per group, also see Figure S1. Four MEG channels containing large amounts of non‐physiological noise were removed from all source‐level analyses. The pre‐processed data were then separated into experimental conditions and downsampled to 200 Hz to aid computation time.

### 2.6 MEG‐MRI Coregistration

MEG data were co‐registered with participants' T1 MRI structural scan by matching the digitised head shape data with surface data from the structural scan (Jenkinson and Smith 2001). Subsequently, the aligned MRI‐MEG image was used to create (1) a forward model based on a single‐shell description of the inner surface of the skull (Nolte 2003), using the segmentation function in SPM8, and (2) spatial normalisation parameters to create individual volumetric grids. To facilitate group analysis, each individual volumetric grid was warped to a template based on the MNI brain, 8‐mm resolution. Subsequently, the inverse of the normalisation parameters were applied to the template grid, for source analysis.

### 2.7 Sensor Level Analysis

Sensor‐level time‐frequency representations (TFRs) were calculated using a single Hanning taper between frequencies of 2–30 Hz in steps of 1 Hz. The entire 2.5‐s epoch was used, with a sliding window of 0.5 s, but the first 0.25 s and last 0.5 s of each trial were discarded to avoid edge artefacts. Due to different scales between the two MEG sensor‐types, only data from the gradiometers were used, with TFR power averaged across each adjoining pair of gradiometers post hoc. All analyses were computed on single trials and subsequently averaged, and therefore TFRs contain both phase‐locked (evoked) and non‐phase‐locked (induced) information.

We focussed our analysis on two frequency bands—theta‐band (3–7 Hz) and alpha (8–12 Hz) power as these have been shown to underly perspective‐taking and perspective‐tracking on the same task (Seymour et al. 2018; Wang et al. 2016). As per our previous analyses (Seymour et al. 2018) we compared TFRs in relation to angular disparity effects (160° vs. 60°) in LR and VO trials separately. Next, due to our hypothesis regarding differing cognitive strategies underlying perspective‐taking in autistic versus non‐autistic participants, we examined TFR differences between groups separately for LR‐160 and LR‐60 trials. Group‐differences related to these contrasts were calculated using cluster‐based permutation testing (see Section 2.9).

### 2.8 MEG Source Level

Source localisation was conducted using Linearly Constrained Minimum Variance beamformer (LCMV, Van Veen et al. 1997) which applies a spatial filter to the MEG data at every voxel of a canonical 0.8‐cm brain grid, in order to maximise signal from that location and attenuating signals elsewhere. The spatial filter was calculated using a covariance matrix from a time–frequency tile centred on the effects found at sensor level. For all analyses, an average filter across baseline (−0.65 to 0 s) and task (0 to 0.65 s) was used. Due to rank reduction following Maxfilter, a regularisation parameter of lambda 5% was applied to the covariance matrix.

For statistical testing, cluster‐based non‐parametric permutation testing was used to correct for multiple comparisons across voxels (Maris and Oostenveld 2007). The resulting whole‐brain statistical maps were presented on a cortical mesh using the Connectome Workbench software (Van Essen et al. 2012). Interpretation of cortical maps was performed with reference to the HCPMMP 1.0 atlas (Glasser et al. 2016).

### 2.9 Statistical Analysis

For MEG data, statistical analysis was performed using cluster‐based permutation tests (Maris and Oostenveld 2007), which consist of two parts: First an independent‐samples *t* test is performed, and values exceeding an uncorrected 5% significance threshold are grouped into clusters. The maximum t‐value within each cluster is carried forward. Second, a null distribution is obtained by randomising the condition label (e.g., autistic/non‐autistic) 5000 times and calculating the largest cluster‐level *t*‐value for each permutation. The maximum *t* value within each original cluster is then compared against this null distribution, and the null hypothesis is rejected if the test statistic exceeds a threshold of *p* < 0.05 (corrected across both tails, i.e., *p* < 0.025 for each tail).

### 2.10 Correlations With Other Measures

We correlated group‐differences in the oscillatory basis of perspective‐taking with various behavioural data from the autistic group: Autism Quotient (Baron‐Cohen et al. 2001); the Glasgow Sensory Questionnaire (Robertson and Simmons 2013); and reaction time from the perspective‐taking behavioural data (LR‐160 condition). The Pearson correlation coefficient was used.

The participants in this study also completed a separate MEG scanning session where they viewed visual gratings. These data were previously reported in Seymour et al. (2019)—we found reduced phase‐amplitude coupling (PAC) and reduced feedback connectivity in autistic participants. To determine links between these two datasets we correlated group‐differences in the oscillatory basis of perspective‐taking with PAC from the visual grating dataset as well as 8–12 Hz directed asymmetry index (DAI), which is a measure of feedback versus feedforward connectivity in the visual system (see Seymour et al. 2019, for more details).

## 3 Results

### 3.1 Behavioural Results

Median reaction times (RT) from each participant, see Figure 1B, were entered into an 2 × 2 × 2 mixed ANOVA, with condition (LR, VO) and angle (160°, 60°) as repeated measures factors and group (ASC, control) as a between‐subjects factor. Results showed a significant interaction between angle and condition on RT, F(1,29) = 29.12, *p* < 0.001, see Figure 1B, replicating previous studies (Kessler and Rutherford 2010; Michelon and Zacks 2006; Seymour et al. 2018; Wang et al. 2016) that reported a significant angular disparity effect for LR judgements (VPT‐2) but not VO judgements (VPT‐1). Post hoc tests revealed that the interaction was indeed due to significantly longer RT for the LR‐160 conditions compared with all other conditions (*p*_tukey_ < 0.001). There was also a main effect of group on RT, *F*(1,29) = 5.68, *p* = 0.024. While there was no statistically significant three‐way interaction (*p* = 0.18), a post hoc analysis showed that the autistic group had longer RT compared with the non‐autistic group, specifically in the LR‐160 condition (*p* = 0.005, all other conditions were *p* > 0.05, see Figure 1B). Accuracy data are reported in Figure S2 and show no significant group differences.

### 3.2 Theta‐Band (3–7 Hz) Results

**FIGURE 2:**
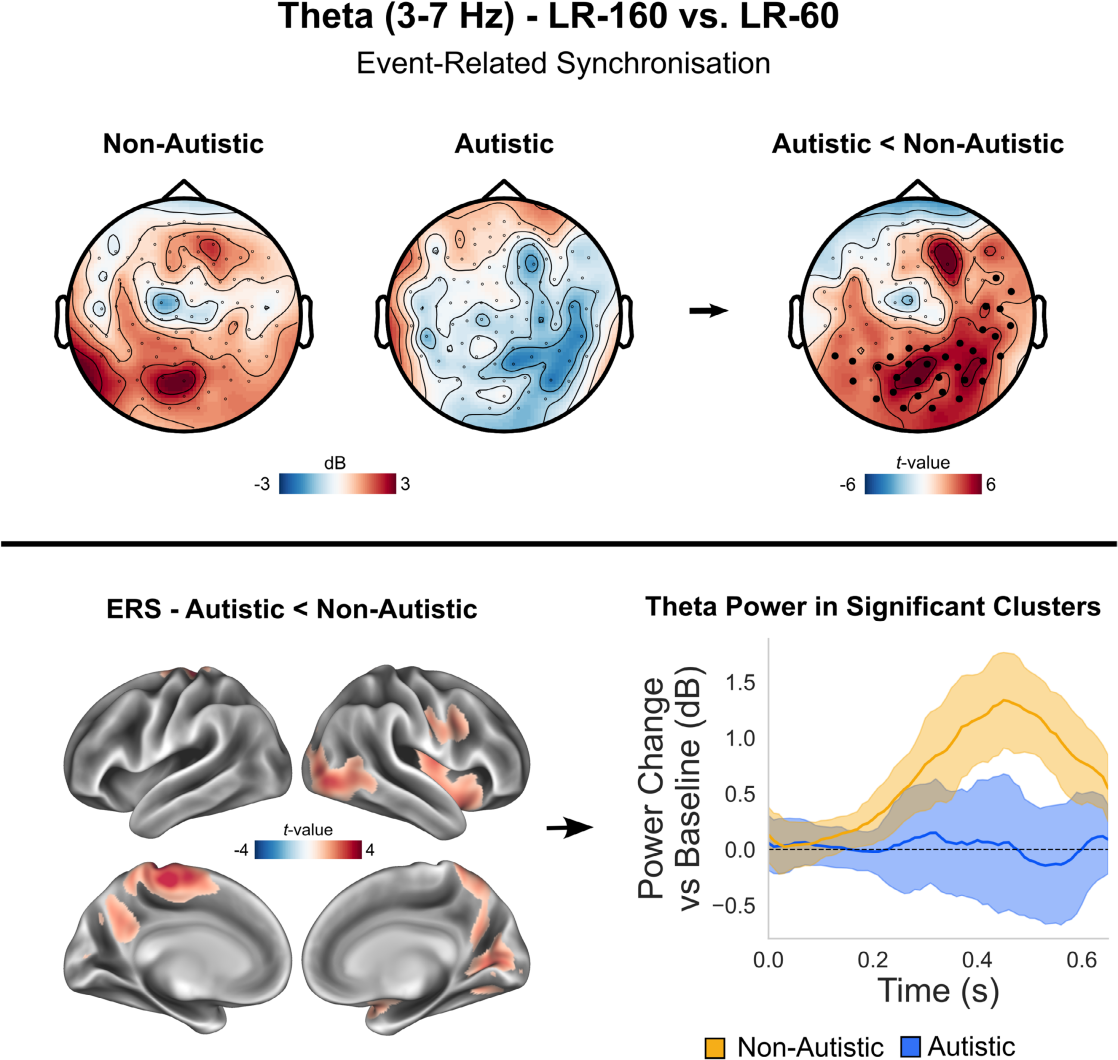
Theta‐band (3–7 Hz) results. In the top panel, sensor‐level topoplots (gradiometers only) are shown for the difference in theta‐band (3–7 Hz, 0–0.65 s) oscillatory power between LR‐160 versus LR‐60 trials. Scales represent MEG field strength, with units of decibels (dB). Statistical comparison of the two groups is shown on the far right, with units of *t* values. Sensors showing a significant difference between groups, *p* < 0.05, are highlighted with a boldened black dot. In the bottom panel the theta‐band group difference source‐localisation results are shown on an inflated cortical mesh, after correcting for multiple comparisons. Theta power was extracted from significant clusters in source space and plotted for non‐autistic (yellow) and autistic group (blue) from 0–0.65 s. Error bars represent 95% confidence intervals.

We first focussed on sensor‐level theta‐band power given previous research showing that embodied perspective‐taking engages a network of brain regions coordinated by theta‐band oscillations (Seymour et al. 2018; Wang et al. 2016). Using the same sensor‐level time‐frequency pipeline as in Seymour et al. (2018) we compared LR‐160 versus LR‐60 trials to investigate the effect of angular disparity during perspective‐taking. This contrast is related to the cognitive effort of embodied perspective‐taking—participants take longer when the avatar's perspective is 160° away from their own perspective, versus 60°. As expected, in the non‐autistic control group there were large increases in theta power (3–7 Hz, 0–0.65 s) in LR‐160 versus LR‐60 trials. However, this was not the case for the ASC group. Statistically comparing 3–7 Hz theta power (0–0.65 s) between groups revealed one significant cluster of greater power in the non‐autistic group at 0.3–0.65 s in the LR‐160 versus LR‐60 condition (*p* = 0.011, max *t* value = 6.03, see Figure 2 top panel).

Next, source localisation was performed to find the brain regions underlying these effects. When contrasting LR‐160 versus LR‐60 trials in the non‐autistic group, increased theta power was found within a widespread collection of regions including ventral visual and occipito‐temporal cortex, right temporo‐parietal and medial parietal cortex, sensorimotor cortices, as well as lateral, medial and ventral prefrontal cortex (see Figure S3). Statistically comparing 3–7 Hz power (0.3–0.65 s) in source‐space revealed that there were group differences in theta power within three clusters (see Figure 2, bottom panel): first centred on left/right sensorimotor cortex (*p* = 0.027, max *t* value = 3.91, max MNI coordinate = [−10, −24, 66]); next over left precuneus, extending into right dorsal visual regions (*p* = 0.041, max *t* value = 3.12, max MNI coordinate = [−12, −64, 34]); and lastly right lateral prefrontal cortex (*p* = 0.039, max *t* value = 3.82, max MNI coordinate = [46, 18, 20]). Theta power was extracted from these significant clusters, averaged and plotted from 0–0.65 s separately for each group (see Figure 2, bottom right). Note the clear group differences in theta power emerge from ~0.3–0.65 s post‐stimulus onset.

In contrast to perspective‐taking (LR trials), perspective‐tracking (VO trials) has previously been shown to rely significantly less on theta‐oscillations (Wang et al. 2016). In line with this, when comparing theta power between VO‐160 versus VO‐60 trials, both groups showed very small increases in theta‐power and no significant group differences were found (see Figure S4).

### 3.3 Alpha‐Band (8–12 Hz) Results

**FIGURE 3:**
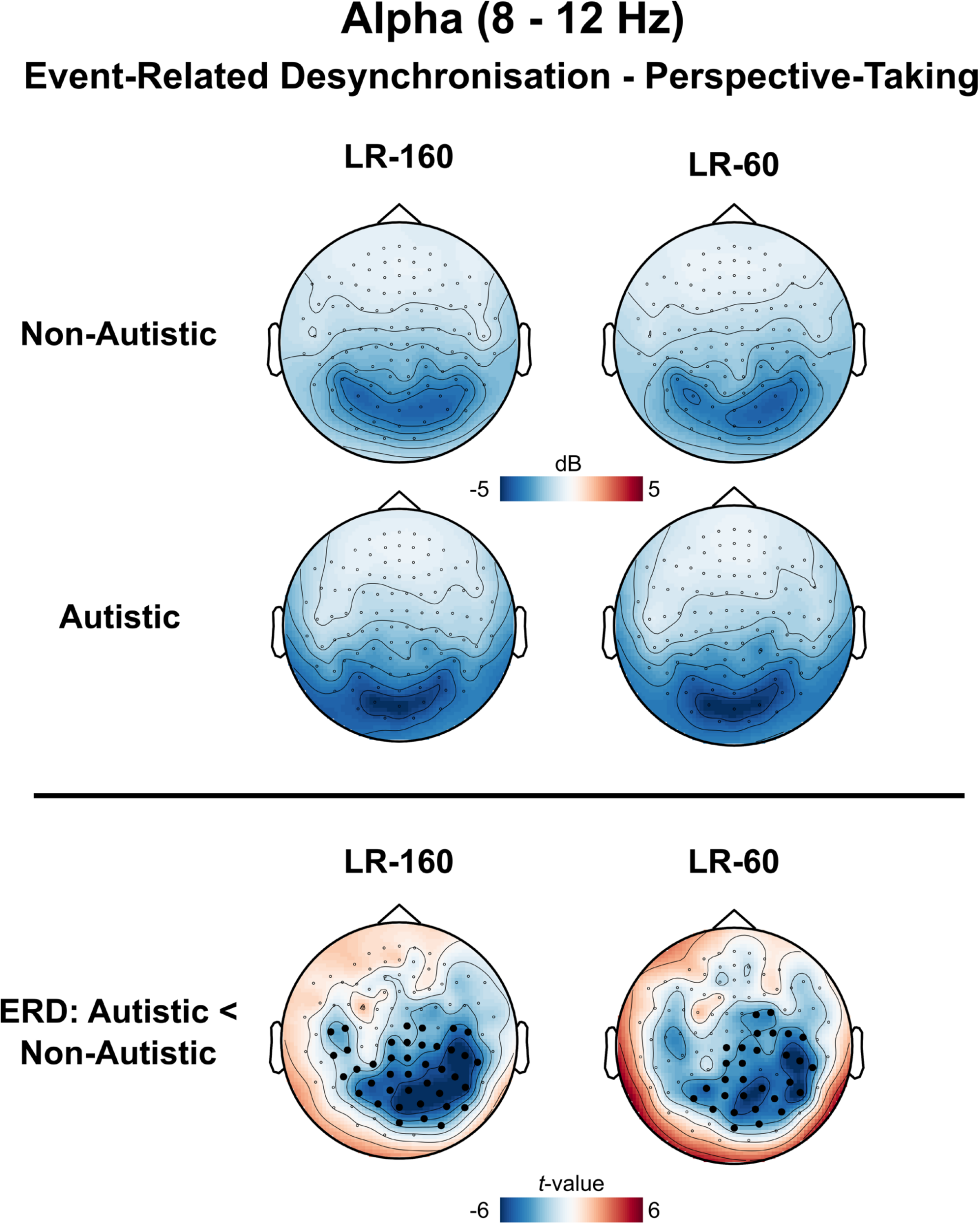
Sensor‐level alpha‐band (8–12 Hz) results. In the top panel, sensor‐level topoplots (gradiometers only) are shown for LR‐160 and LR‐60 trials (8–12 Hz, 0–0.8 s). Scale represents MEG field strength, baseline‐corrected, with units of decibels (dB). Statistical comparison of the two groups is shown in the bottom panel for LR‐160 and LR‐60 trials separately, with units of t‐values, with sensors showing a significant difference, *p* < 0.05, highlighted with a boldened black dot.

Sensor‐level TFRs were calculated using the same pipeline as in Seymour et al. (2018). For both the ASC and control groups perspective‐taking was accompanied by large alpha‐band (8–12 Hz) desynchronization over posterior sensors (see Figure 3) in both the LR‐160 and LR‐60 trials. Statistically comparing groups in the alpha band, we found that the autistic group had greater alpha‐desynchronization over posterior sensors in both LR‐160 (cluster times = 0.19–0.82 s, *p* = 0.025, max *t* value = 5.89) and LR‐60 trials (cluster times: 0.23–0.76 s, *p* = 0.034, max *t* value = 5.23).

Next, we performed a post‐hoc comparison of LR‐160 versus LR‐60 alpha power at the time‐points identified in the above analysis. However, unlike Wang et al. (2016), we did not observe a statistical difference in alpha‐band power when comparing LR‐160 versus LR‐60 trials in either group, *p* = 0.89. Additionally, an ANOVA showed no group x condition interaction effects, *p* = 0.94. The above analyses were also repeated for perspective‐tracking (i.e., VO trials); however, no significant group differences, were found for alpha‐band power (LR‐160: *p* = 0.87; LR‐60: *p* = 0.62).

**FIGURE 4:**
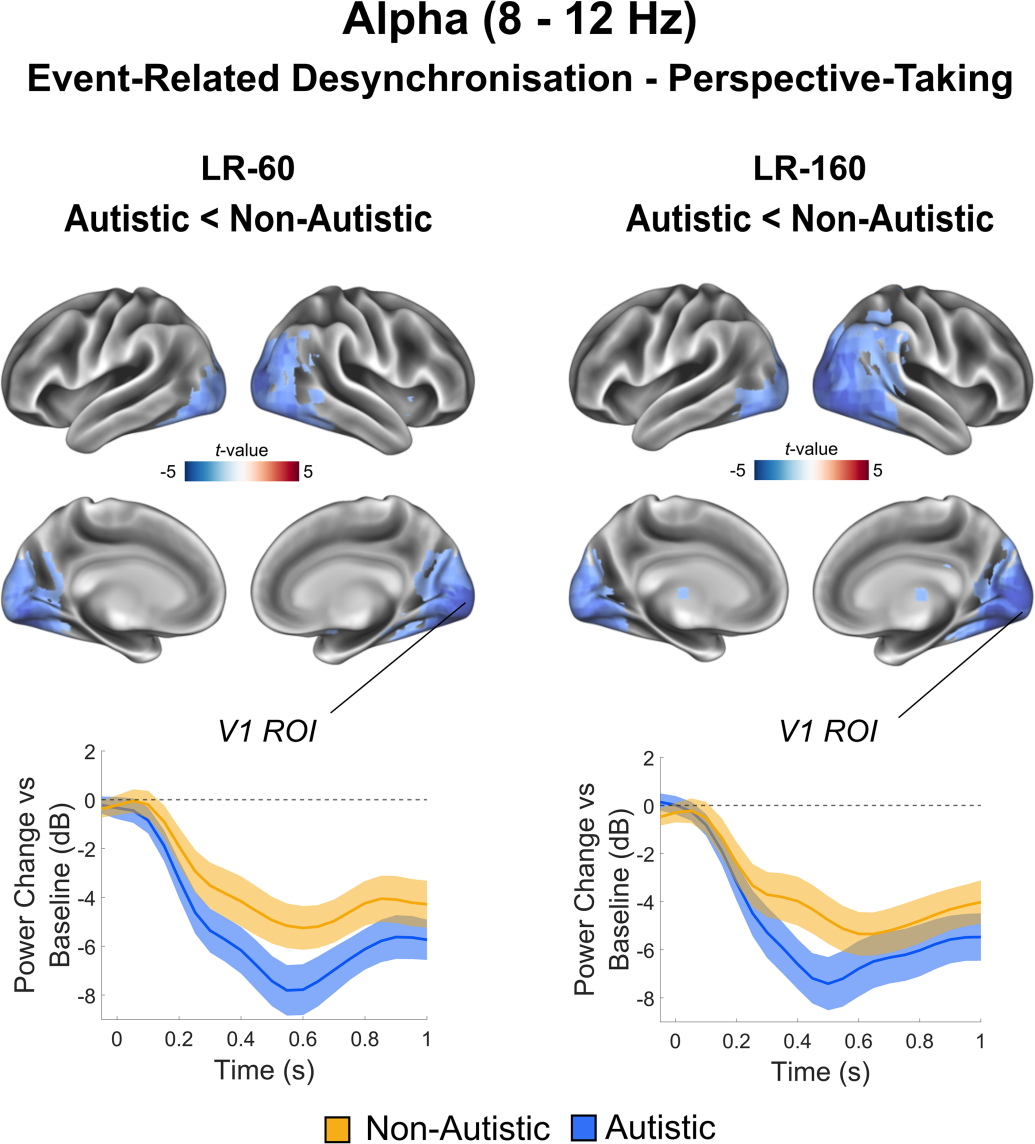
Source‐level alpha‐band (8–12 Hz) results. Alpha‐band power (8–12 Hz, 0–0.8 s) was localised using a beamformer. In the top panel baseline corrected alpha power results are plotted for each condition (LR‐160, LR‐60) and group (autistic, non‐autistic). Only clusters passing a *p* < 0.05 corrected threshold are shown. A region of interest was defined in primary visual cortex (V1) and the alpha power change versus baseline was calculated for LR‐160 and LR‐60 trials. The bottom panel plots this in a time‐resolved manner separately for non‐autistic (yellow) and autistic groups (blue). Error bars correspond to 95% confidence intervals.

Using the same source‐localisation pipeline as for theta and timepoints identified in the sensor‐level analyses (see Figure 3, bottom panel), we investigated the cortical sources underlying 8–12 Hz alpha desynchronisation in perspective‐taking. As expected, the group difference in alpha power localised to occipital cortex, with a peak in primary visual cortex, in both LR‐160 (*p* = 0.009, max *t* value = 5.10, max MNI coordinate = [−22, −94,3]) and LR‐60 trials (*p* = 0.013, max *t* value = 4.98, max MNI coordinate = [−33, −82,9]), see Figure 4, top panel. To investigate the temporal progression of alpha‐related group differences in further detail we extracted virtual time‐series from a region of interest in primary visual cortex. In both LR‐160 and LR‐60 trials the autistic group (yellow line) displayed larger alpha desynchronisation in LR‐60 trials from 0.31 to 1.0 s, *p* = 0.004, and for LR‐160 trials from 0.36 s to 0.61 s, *p* = 0.036, post‐stimulus presentation compared with the non‐autistic group (blue line), see Figure 4, bottom panel.

### 3.4 Correlations With Other Measures

Finally, we investigated correlations between our neural findings in the theta/alpha‐bands and various measures from the autistic group (see Section 2). Specifically, for each autistic participant we extracted data from sensors showing a significant group difference versus the non‐autistic control group in the theta band (3–7 Hz, 0.3–0.65 s) and alpha band (8–12 Hz, 0–0.8 s). Only one significant correlation was found—between alpha power desynchronization and the directed asymmetry index of alpha in the visual stream (DAI), *r* = 0.512, *p* = 0.043, which represents a measure of feedback connectivity as reported by Seymour et al. (2019). These data are shown in Figure S5. No other correlations were significant, *p* > 0.05, including data from the AQ and GSQ questionnaires.

## 4 Discussion

In this study, we investigated the neural oscillatory basis of high‐level perspective‐taking in a group of autistic adolescents and a non‐autistic group of age‐matched control participants. The paradigm was specifically designed to separate perspective‐tracking (‘can the other person see the target or not?’) from perspective‐taking (‘is the target to the other person's left or right?’). Behaviourally, the autistic group had longer reaction times compared with age‐matched controls, specifically for perspective‐*taking* trials in which the target's perspective was 160° versus 60° away from the participant's own perspective. No group differences were found for perspective‐*tracking* trials. This supports the notion of a selective difference regarding embodied strategies during perspective‐taking but not tracking in ASC (Pearson et al. 2013, 2016). Using MEG, we further showed that when increasing the angular disparity between self and other perspectives (160° vs. 60°), non‐autistic adolescent participants showed increased theta‐band (3–7 Hz) power. This theta pattern was absent in the autistic group and a direct comparison in source space revealed contrasting theta power differences within ventral visual and occipito‐temporal cortex, medial parietal cortex, sensorimotor cortices and lateral and ventral prefrontal cortex. However, when examining alpha rhythms a very different picture emerged. Here, there were no group differences when the angular disparity increased from 60° to 160°. Instead, across both LR‐160 and LR‐60 trials the autistic group showed greater alpha desynchronisation over occipital cortices from 0.3 s after stimulus onset compared with the non‐autistic group.

When examining perspective‐tracking (VPT‐1) trials, for both, theta and alpha‐bands no significant differences between groups were observed, corroborating the null result in behavioural data and the view that autistic and non‐autistic individuals do not appear to differ in their strategy for perspective‐tracking (Pearson et al. 2013).

The very distinct patterns in oscillatory signatures observed for the two groups during perspective‐taking (VPT‐2), that is, theta versus alpha effects, are more likely to reflect differences in cognitive strategies (Gardner et al. 2013; Kessler and Wang 2012; Pearson et al. 2013, 2016; Samuel et al. 2023) than a domain‐general deficit in cognitive control in autism (e.g., Rajendran and Mitchell 2007, for review). The latter predicted similar but stronger theta and alpha signatures alongside poorer performance (longer RTs and more mistakes) in the autistic compared to the non‐autistic group.

Stronger theta power in the non‐autistic group localised to ventral visual and occipito‐temporal cortex, right temporo‐parietal and medial parietal cortex, sensorimotor cortices, as well as lateral, medial and ventral prefrontal cortex. This replicates previous MEG studies (Seymour et al. 2018; Wang et al. 2016) showing increased theta power in a similar set of brain regions during perspective‐taking, coordinated by the right TPJ. We believe this pattern of brain network activation in the non‐autistic control group, especially theta activity within right TPJ and sensorimotor cortex, reflects the use of an embodied strategy (Kessler and Thomson 2010; Wang et al. 2016; Gooding‐Williams et al. 2017). The involvement of medial and lateral prefrontal regions is thought to manage conflict between self and other perspectives, which has been shown to be affected in autism (Carrington and Bailey 2009; Conson et al. 2015; Hartwright et al. 2012). Our findings imply that autistic adolescents are less able to, or simply less inclined to use such an embodied mental self‐rotation strategy.

The perspective‐taking paradigm used in this study can, however, be solved in another way—through mental object rotation (Kessler and Thomson 2010; Samuel et al. 2023; Zacks and Tversky 2005). Alternatively, autistic participants might favour a visuospatial strategy where the other's left and right are still aligned with the egocentric left and right at low angular disparity (60°), allowing for a very quick left/right decision, while at high angular disparity (160°, i.e., almost face‐to face) their egocentric left would be the other's right and their right the other's left, only requiring a visuospatial transposition (e.g., Gardner et al. 2013). Our finding of stronger posterior alpha desynchronization hints at the possibility that the autistic group were using one of these alternative strategies to solve the task (Conson et al. 2015; Pearson et al. 2016). Posterior alpha power desynchronisation reflects reduced functional inhibition during visual perception (Jensen and Mazaheri 2010), suggesting a far more sensory, bottom‐up manipulation of the visual stimuli in the ASC group. In other words, autistic participants may favour manipulating the visual percept rather than relying on an embodied, social cognitive strategy favoured by non‐autistic participants. Interestingly, we found a significant negative correlation between alpha desynchronisation and the amount of V4‐to‐V1 feedback connectivity as measured during a complimentary low‐level visual task in the same participants (Seymour et al. 2019). These findings imply that when ASC participants are able to use a visual or object‐rotation strategy they do so, but at the detriment of the more efficient and socially engaged strategy of embodying another's perspective through mental self‐rotation into their viewpoint. In support of an object‐rotation strategy, previous research using TMS (Klimesch et al. 2003), TACS (Kasten and Herrmann 2017) and neurofeedback (Hanslmayr et al. 2005) have shown that alpha‐band oscillations are causally linked with mental object rotation abilities. Interestingly, our alpha desynchronisation group difference did not interact with angular disparity—no effect of alpha power was found when comparing trials where the object was 160° versus 60° from the participant's perspective. This implies that the same amount of visual processing was engaged by the autistic group despite the 100° difference in rotation required. This could suggest that a visuospatial transposition strategy explanation might be likely (‘my left is their right’ at 160°) or that the stimuli in this study required only very simple mental rotations along just one dimension rather than true three‐dimensional object rotation as often used with Shepard and Metzler type stimuli (Shepard and Metzler 1988).

More generally, our findings underscore the importance of studying different cognitive strategies in perspective‐taking (Kessler et al. 2014; Samuel et al. 2023, 2024). Alpha desynchronisation is likely to be a neural signature of effortful and slower object‐rotation strategy or indicative of a visuospatial transposition strategy (e.g., ‘spatial transposers’ Gardner et al. 2013; also Kessler and Wang 2012), whereas increases in theta power associated with the faster and more embodied self‐rotation strategy in controls (Wang et al. 2016). Future research could investigate this further by characterising the neural dynamics of embodied processing and mental rotation within the same participants. Repetitive TMS entrainment could also be used to investigate the causal role of alpha vs theta oscillations as a function of neurocognitive strategy during perspective‐taking (Gooding‐Williams et al. 2017).

Finally, our results show that when instructed to imagine the world from another's perspective, autistic participants activate visual regions rather than the extended network of regions supporting embodied transformation, including temporal, parietal, sensorimotor, and prefrontal areas (Wang et al. 2016; Seymour et al. 2018). A bias towards prioritising bottom‐up sensory information, at the expense of top‐down prior information, has been described by predictive‐coding accounts of autism (Kessler et al. 2016; Palmer et al. 2017; Pellicano and Burr 2012). These generally focus on sensory aspects of ASC but can be easily extended for social and embodied processes (e.g., Kessler et al. 2016). Just as one can represent an object for perceptual inference, one can also represent another's perspective, or mental state, if it helps to predict the causes of sensory input and minimise prediction error (Palmer et al. 2015). Embodied processing may be particularly affected in autism as another's perspective is abstractly inferred, presumably involving top‐down mechanisms and regions at the top of the cortical hierarchy (Friston 2011). Our findings hint at the intriguing possibility of a common mechanism underlying both sensory and social symptoms in autism. Impaired embodied abilities may result in cascading deficits or atypicality in the development of social cognitive skills like Theory of Mind (Kessler et al. 2016; Pearson et al. 2013). While the paradigm in this study required a relatively simple spatial judgement (left/right), there is evidence to suggest that visual perspective‐taking performance can predict theory of mind in children (Hamilton et al. 2009). For perspective‐taking, autistic individuals can adopt alternative compensatory strategies, potentially supported by increased alpha desynchronisation over visual cortex (Hanslmayr et al. 2005; Kasten and Herrmann 2017; Klimesch et al. 2003; Pearson et al. 2016). In fact, the egocentric nature of these alternatives might appeal to autistic individuals, since a heightened egocentric bias has been proposed in autism (e.g., Baron‐Cohen 1997; Lombardo and Baron‐Cohen 2010, 2011). However, as tasks become more abstract, for example, false belief tasks requiring representing what others are thinking (Yuk et al. 2020), these strategies become inefficient, if not impossible. We note three limitations. First, our assertion that alpha desynchronisation reflects mental rotation strategy relies on reverse inference. However, there are both theoretical (Pearson et al. 2013, 2016; Samuel et al. 2023, 2024) and quantitative grounds (Gardony et al. 2017; Hanslmayr et al. 2005; Kasten and Herrmann 2017; Zacks and Michelon 2005) to support this. To further our argument, future MEG studies could explicitly ask participants about which strategies they are using, and/or measure mental rotation alongside perspective‐taking in the same participants. Second, we did not collect a formal clinical assessment of autism, for example, the Autism Diagnostic Observation Schedule (Lord et al. 2000), to ascertain symptom severity. Instead, strict participant exclusion criteria were implemented, and only autistic participants with a confirmed clinical diagnosis of Autism Spectrum Disorder or Asperger's syndrome were included. Between groups, there were significant differences in autistic and sensory traits, see Table 1. Future research with larger cohorts of autistic individuals (across a wider age range) would be beneficial for determining how perspective‐taking and embodiment interacts with the development of language acquisition, theory of mind and sensory sensitivities in autistic individuals. Additionally, larger sample sizes would help clarify how sex differences influence perspective‐taking strategies in autistic individuals. Finally, we note that based on the current findings, we cannot disambiguate between a mental rotation and a visuospatial transposition strategy. Future research should aim to understand in more detail what strategy is preferably engaged by autistic participants through self‐report questionnaires.

While our interpretation of the perspective‐taking differences has focussed on embodiment, it is important to acknowledge that domain‐general explanations of autism have been proposed (see Rajendran and Mitchell 2007, for review). These theories suggest that executive function and cognitive control are at the heart of ASD. However, in this study we did not observe behavioural accuracy differences between the autistic and non‐autistic group indicative of reduced executive control. Furthermore, we observed different frequency modulations between the groups, suggesting the use of different strategies, rather than general reductions in embodied theta‐band processing. In other words, autistic participants can take the perspective of others, just in a different way. Our data are therefore more consistent with specific social and cognitive differences in autism, perhaps related to self‐other distinctions (Lombardo et al. 2011), rather than a domain general executive differences.

More generally, we suggest that disambiguating different perspective‐taking strategies in autism could not only shed light on basic social cognition (Samuel et al. 2024) but also inform the development of theory‐led interventions. Impaired visual perspective‐taking has been linked to difficulties in social skill development in autism (Hamilton et al. 2009; Pearson et al. 2016; Surtees et al. 2013), raising the intriguing possibility that interventions targeting perspective‐taking could yield broader benefits for high‐level social and cognitive development (Pearson et al. 2016). For example, embodied mental transformations also plays a key role in broader spatial abilities such as motor learning and navigation (Hegarty and Waller 2004; Kozhevnikov et al. 2006). Expanding the repertoire of embodied transformations while decreasing reliance on egocentric strategies may have wide‐reaching benefits for autistic individuals.

## 5 Conclusion

Using MEG, we show that the neural basis of visual perspective‐taking differs between autistic adolescents and non‐autistic age‐matched controls. Behaviourally, autistic participants took longer to respond, specifically when the angle between self and other perspectives was high. Neural processing in the autistic group was dominated by alpha‐related signatures in the visual system. In contrast, the control group showed strong neural responses within an extended theta‐band network of temporo‐parietal and pre‐frontal regions required for embodied transformation. We argue this represents a split strategy between the groups, with autistic adolescents appearing to favour a visuospatial transposition or mental object rotation strategy, whereas non‐autistic adolescents seem to favour an embodied self‐rotation strategy, similar to non‐autistic adults (Kessler and Rutherford 2010; Seymour et al. 2018; Wang et al. 2016). Our findings also support claims that autism is solely associated with differences in perspective‐taking (VPT‐2) rather than tracking (VPT‐1) (Pearson et al. 2013, 2016).

## Author Contributions

**Robert A. Seymour:** conceptualization, data curation, formal analysis, investigation, methodology, project administration, resources, software, validation, visualization, writing – original draft, writing – review and editing. **Gina Rippon:** funding acquisition, supervision, writing – review and editing. **Hongfang Wang:** data curation, methodology, software. **Gerard Gooding‐Williams:** data curation, investigation, writing – review and editing. **Klaus Kessler:** funding acquisition, resources, supervision, writing – review and editing.

## Conflicts of Interest

The authors declare no conflicts of interest.

### Peer Review

The peer review history for this article is available at https://www.webofscience.com/api/gateway/wos/peer‐review/10.1111/ejn.70109.

## Supporting information


***Supplementary Figure 1*** The number of ‘good’ trials after removing trials with incorrect responses and trials containing artefacts was calculated for each condition and for each group. Results of a 2x2x2 mixed ANOVA showed that there were no significant differences in accuracy between the groups, F(1,29) = 0.08, *p* = 0.780.
***Supplementary Figure 2:*** The percentage of correct responses was calculated for each condition and for each group. Overall accuracy was very high. Results of a 2x2x2 mixed ANOVA showed that there were no significant differences in accuracy between the groups F(1,29) = 0.40, *p* = 0.530.
***Supplementary Figure 3:*** Perspective‐taking theta source localisation results. For the non‐autistic control group, we localised theta power (3–7 Hz) and statistically compared LR‐160 versus LR‐60 trials (0–0.65 s). Power maps are presented on an inflated brain from HCP Workbench. Only clusters passing a *p* < 0.05 threshold, corrected for multiple comparisons, are shown. In replication of Wang et al. (2016) and Seymour et al. (2018), significant clusters are observed in right posterior temporo‐parietal junction, right visual ventral stream, right prefrontal cortex, and in medial cortical areas such as the cingulate cortex (ranging from posterior to anterior.
***Supplementary Figure 4:*** We investigated theta‐power during perspective‐tracking where participants were asked to judge if the target was visible or occluded from the avatar’s perspective. Sensor‐level time‐frequency representations were calculated using the same pipeline as outlined in the main manuscript. Paralleling the perspective‐taking analysis, we compared theta power (3–7 Hz, 0–0.65 s) in VO‐160 vs. VO‐60 trials. Overall there were very small changes in theta power and no significant group differences for perspective‐tracking.
***Supplementary Figure 5:*** In the autistic group a significant correlation, r = 0.512, *p* = 0.043, was found between the amount of alpha‐band synchronisation during perspective‐taking and the directed asymmetry index (DAI) reported in Seymour et al. (2019) which represents a measure of alpha‐band V4‐to‐V1 feedback connectivity in the visual system. In this instance, the lower the DAI value the greater the amount of feedback connectivity in the visual system, which correlates with the amount of alpha desynchronisation observed in the perspective‐taking task.

## Data Availability

The data that support the findings of this study are available on reasonable request from corresponding author, R.S., in a preprocessed and de‐anonymized form. The raw data are not publicly available due to ethical restrictions. MATLAB data analysis code for this study is available from https://github.com/neurofractal/ASD_PT_MEG.
