# Supplementary material for: The Neural Oscillatory Basis of Perspective‐Taking in Autistic and Non‐Autistic Adolescents Using Magnetoencephalography
Source: Eur J Neurosci. 2025 Apr 16;61(8):e70109. doi: 10.1111/ejn.70109 (PMC12001870; doi:10.1111/ejn.70109)
Supplement: Supplementary file 1 — Supplementary Figure 1 The number of ‘good’ trials after removing trials with incorrect responses and trials containing artefacts was calculated for each condition and for each group. Results of a 2x2x2 mixed ANOVA showed that there were no significant differences in accuracy between the groups, F(1,29) = 0.08, p = 0.780. Supplementary Figure 2: The percentage of correct responses was calculated for each condition and for each group. Overall accuracy was very high. Results of a 2x2x2 mixed ANOVA showed that there were no significant differences in accuracy between the groups F(1,29) = 0.40, p = 0.530. Supplementary Figure 3: Perspective‐taking theta source localisation results. For the non‐autistic control group, we localised theta power (3–7 Hz) and statistically compared LR‐160 versus LR‐60 trials (0–0.65 s). Power maps are presented on an inflated brain from HCP Workbench. Only clusters passing a p < 0.05 threshold, corrected for multiple comparisons, are shown. In replication of Wang et al. (2016) and Seymour et al. (2018), significant clusters are observed in right posterior temporo‐parietal junction, right visual ventral stream, right prefrontal cortex, and in medial cortical areas such as the cingulate cortex (ranging from posterior to anterior. Supplementary Figure 4: We investigated theta‐power during perspective‐tracking where participants were asked to judge if the target was visible or occluded from the avatar’s perspective. Sensor‐level time‐frequency representations were calculated using the same pipeline as outlined in the main manuscript. Paralleling the perspective‐taking analysis, we compared theta power (3–7 Hz, 0–0.65 s) in VO‐160 vs. VO‐60 trials. Overall there were very small changes in theta power and no significant group differences for perspective‐tracking. Supplementary Figure 5: In the autistic group a significant correlation, r = 0.512, p = 0.043, was found between the amount of alpha‐band synchronisation during perspective‐taking and the [file EJN-61-0-s001.docx]

**Supplementary Materials**

The Neural Oscillatory Basis of Perspective-Taking in Autistic and non-Autistic Adolescents using MEG – *Seymour et al., (2025)*


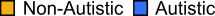


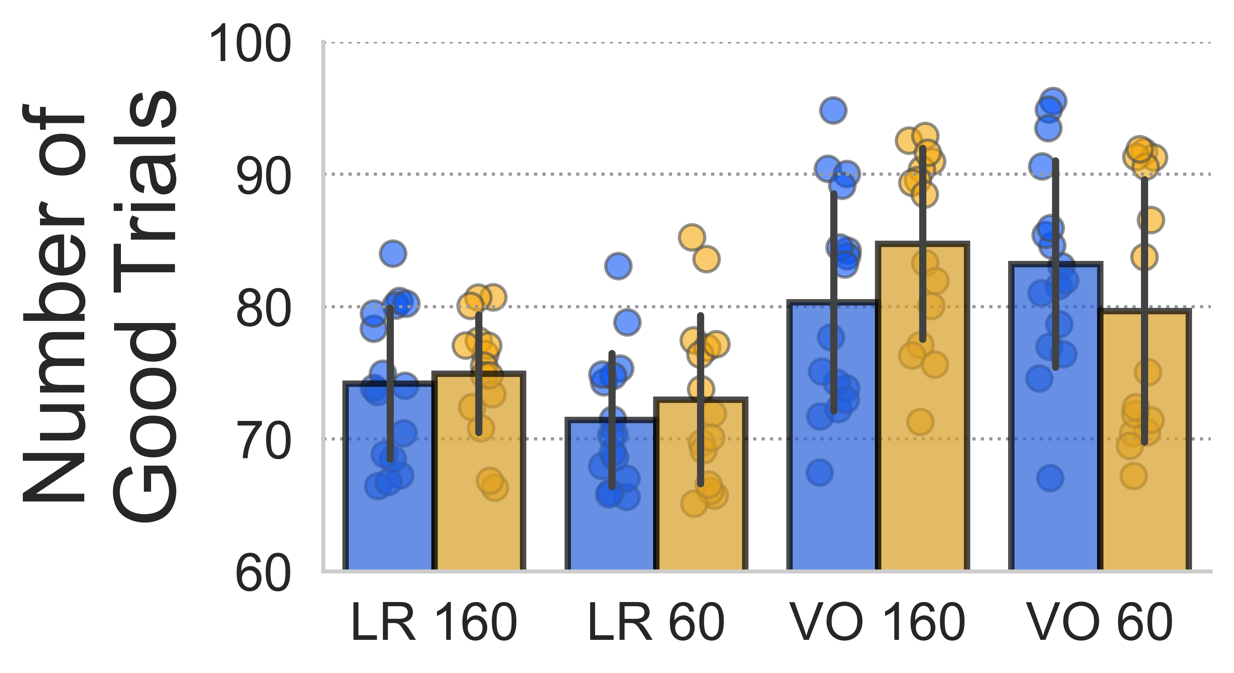


***Supplementary Figure 1:*** The number of ‘good’ trials after removing trials with incorrect responses and trials containing artefacts was calculated for each condition and for each group. Results of a 2x2x2 mixed ANOVA showed that there were no significant differences in accuracy between the groups, F(1,29) = 0.08, p = 0.780.


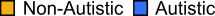


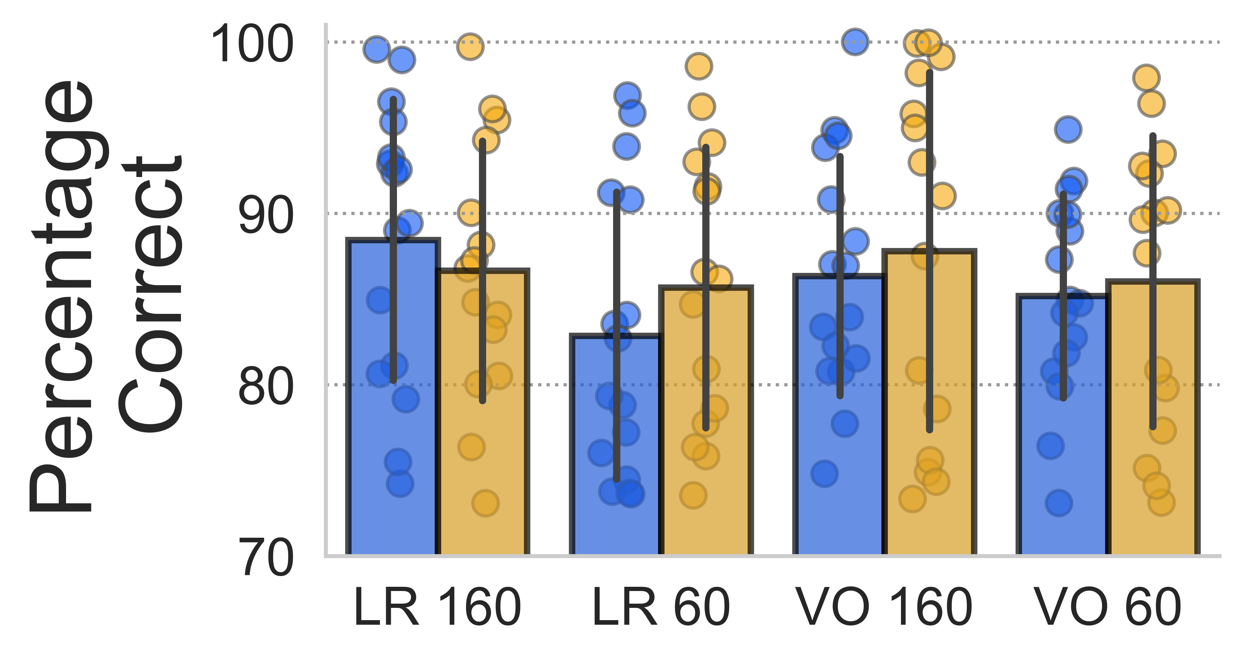


***Supplementary Figure 2:*** The percentage of correct responses was calculated for each condition and for each group. Overall accuracy was very high. Results of a 2x2x2 mixed ANOVA showed that there were no significant differences in accuracy between the groups F(1,29) = 0.40, p = 0.530.


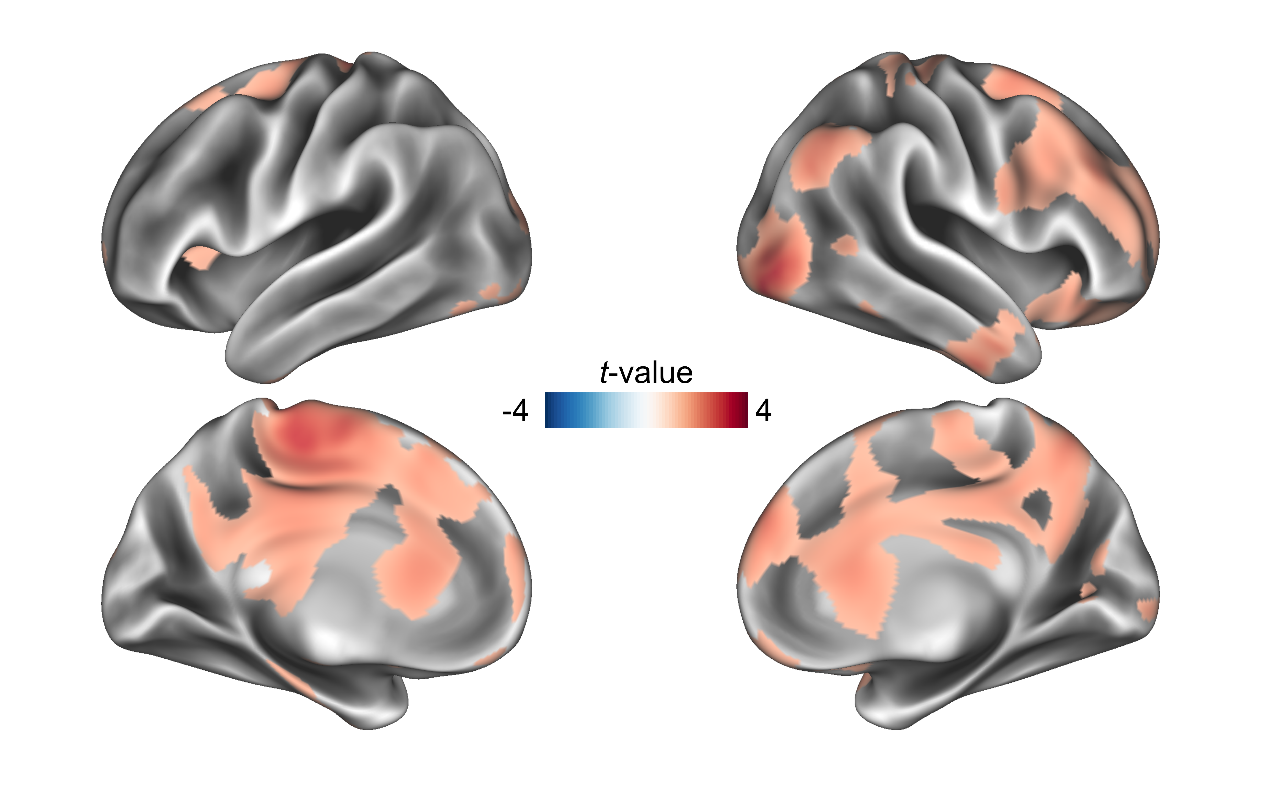


***Supplementary Figure 3:*** Perspective-taking theta source localisation results. For the non-autistic control group, we localised theta power (3-7 Hz) and statistically compared LR-160 versus LR-60 trials (0-0.65 s). Power maps are presented on an inflated brain from HCP Workbench. Only clusters passing a p<.05 threshold, corrected for multiple comparisons, are shown. In replication of Wang et al (2016) and Seymour et al (2018), significant clusters are observed in right posterior temporo-parietal junction, right visual ventral stream, right prefrontal cortex, and in medial cortical areas such as the cingulate cortex (ranging from posterior to anterior.


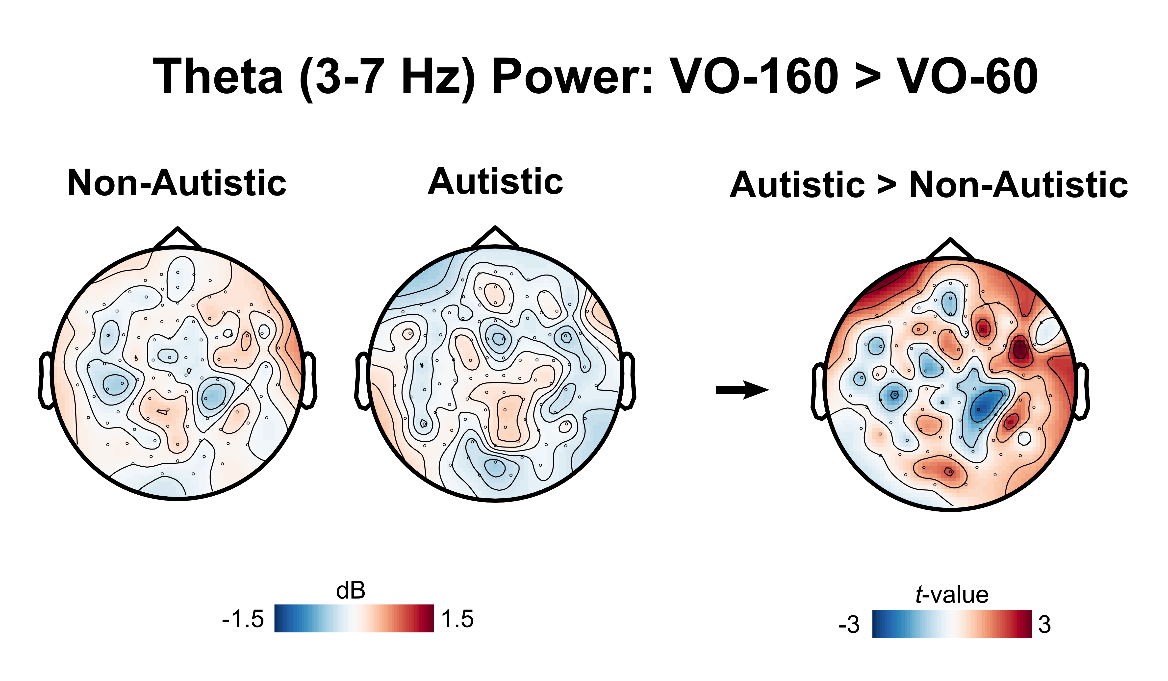


***Supplementary Figure 4:*** We investigated theta-power during perspective-tracking where participants were asked to judge if the target was visible or occluded from the avatar’s perspective. Sensor-level time-frequency representations were calculated using the same pipeline as outlined in the main manuscript. Paralleling the perspective-taking analysis, we compared theta power (3-7 Hz, 0-0.65s) in VO-160 vs. VO-60 trials. Overall there were very small changes in theta power and no significant group differences for perspective-tracking.


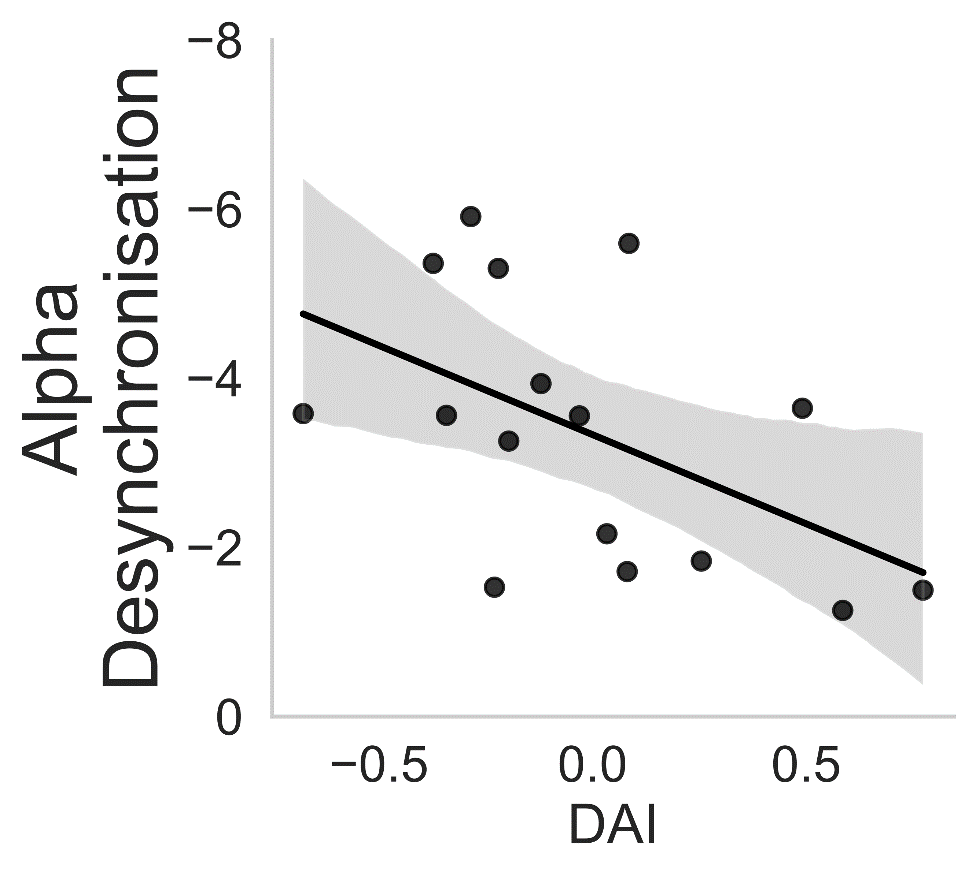


***Supplementary Figure 5:*** In the autistic group a significant correlation, r = .512, p = .043, was found between the amount of alpha-band synchronisation during perspective-taking and the directed asymmetry index (DAI) reported in Seymour et al., (2019) which represents a measure of alpha-band V4-to-V1 feedback connectivity in the visual system. In this instance, the lower the DAI value the greater the amount of feedback connectivity in the visual system, which correlates with the amount of alpha desynchronisation observed in the perspective taking task.
